# What causes high costs for rural tuberculosis inpatients? Evidence from five counties in China

**DOI:** 10.1186/s12879-020-05235-9

**Published:** 2020-07-11

**Authors:** Haomiao Li, Bin Cheng, Yingchun Chen

**Affiliations:** 1 Department of Cardiology, Renmin Hospital of Wuhan University, Wuhan, 430060 China; 2 Institute of Model Animals of Wuhan University, Wuhan, 430072 China; 3 Research center for Rural Health Services, Hubei Province Key Research Institute of Humanities and Social Sciences, Wuhan, 430030 Hubei China; 4 China National Health Development Research Center, Beijing, 100044 China; 5 School of Medicine and Health Management, Tongji Medical College, Huazhong University of Science and Technology, Wuhan, 430030 Hubei China

**Keywords:** Tuberculosis, Rural China, Economic burden, Generalized linear models, Quantile regression, Socio-demographic status, Institutional attributes

## Abstract

**Background:**

Tuberculosis (TB) still causes high economic burden on patients in China, especially for rural patients. Our study aims to explore the risk factors associated with the high costs for TB inpatients in rural China from the aspects of inpatients’ socio-demographic and institutional attributes.

**Methods:**

Generalized linear models were utilized to investigate the factors associated with TB inpatients’ total costs and out-of-pocket (OOP) expenditures. Quantile regression (QR) models were applied to explore the effect of each factor across the different costs range and identify the risk factors of high costs.

**Results:**

TB inpatients with long length of stay and who receive hospitalization services cross provincially, in tertiary and specialized hospitals were likely to face high total costs and OOP expenditures. QR models showed that high total costs occurred in Dingyuan and Funan Counties, but they were not accompanied by high OOP expenditures.

**Conclusions:**

Early diagnosis, standard treatment and control of drug-resistant TB are still awaiting for more efforts from the government. TB inpatients should obtain medical services from appropriate hospitals. The diagnosis and treatment process of TB should be standardized across all designated medical institutions. Furthermore, the reimbursement policy for migrant workers who suffered from TB should be ameliorated.

## Background

Tuberculosis (TB) is the world’s ninth leading cause of death and the leading cause of death from a single pathogen, that is, higher than HIV/AIDS [1]. At present, numerous countries have realized inspiring progress in preventing and treating TB, and the target of the Millennium Development Goals to halt and reverse TB incidence has been achieved worldwide. However, the decrease in TB incidence has been very slow, with an estimated decrease of 2% per year, while approximately 10.4 million new cases and 1.7 million deaths worldwide occurred in 2016 [2]. To reduce the continuing unacceptable burden of TB, the WHO End TB Strategy was adopted by the World Health Assembly in May 2014 and sets out the interventions needed to end the global TB epidemic by 2035. This strategy places a great emphasis on the prevention and care of TB by addressing its social determinants, including policies to alleviate poverty and social protection programs.

China is one of the countries that suffer most from TB, which accounts for 64% of the global burden together with India, Indonesia, the Philippines, Pakistan, Nigeria, and South Africa. According to the WHO TB report in 2017, China had 783,842 TB cases out of its 1.4 billion population in 2016. In a study investigating the basic information of the hospital for tuberculosis (TB) control of China, hospitals with more than 30 beds in the 31 provinces (not including Taiwan, Hongkong and Macao) and Xinjiang Production and Construction Corps were taken into analysis. In 2009, there were 2.5 million TB outpatients and 1.3 million TB inpatients. The average length of stay (LOS) for TB inpatients was 27 days, and the average hospitalization expense was 6631 RMB (1023 USD) [3]. The hospitalization services of TB cost a lot of medical resources for diagnosis, treatment and management, which bring about great economic burden to TB inpatients, especially to rural TB inpatients [4, 5]. Despite the implementation of preferential policies for rural TB patients when obtaining medical services, costs for diagnosis and treatment remain expensive [6, 7] Furthermore, the poor have higher TB prevalence and incidence and face more serious non-medical costs than relatively wealthier patients [8, 9]. Such condition exposes rural TB patients’ households at a higher risk of catastrophic health expenditure. Thus, identifying appropriate ways to reduce rural TB patients’ economic burden effectively is essential and urgent.

These years, China has made great efforts on tuberculosis prevention and cure in rural areas, as well as payment system optimization of medical insurance for TB patients. For example, Yichang City, Hubei Province carried out an exploration on the mode of single disease payment of the New Rural Cooperative Medical System (NRCMS) for TB outpatients and inpatients, in order to improve the reimbursement rate of TB patients by 80% ~ 90%, and reduce the medical burden of tuberculosis patients [10, 11].

The compensation scheme of the NRCMS has been proven to reduce the economic burden of TB patients [12]. However, the effect of NRCMS was modest and the equity did not improve. Prior studies showed that age, household size, employment status, health insurance status, patient income as a percentage of total household income, hospitalization, and status as a minimum living security household are regarded as factors associated with the effect of NRCMS on TB patients’ economic burden [13, 14]. These studies paid further attention to the relations between TB patients’ costs or benefits from medical insurance and their socio-demographic status. However, TB patients’ selections on medical institutions are also essential factors related to their costs or benefits, which are under-researched. The internal management system, service capability of medical institutions, and payment system of medical insurance could affect the treatment costs and direct expenses (out of pocket [OOP]) of TB patients [15, 16]. The patients’ selections of institutions can not only derive from their active awareness, such as the preference for high quality, good hospital environment, or low expenses, but also can be passive, such as the nearby medical treatment for migrant workers [17]. Moreover, when exploring the influencing factors, numerous studies have concentrated on the average costs of TB, rather than segmented analysis, especially the factors related to high costs.

The objective of this study was to explore the impacting factors related to high costs of rural tuberculosis patients in China. As hospitalization services cost more than outpatient services, inpatients were selected as the study population. Socio-demographic characteristics of the TB inpatients and attributes of medical institutions were included. In addition, we analyzed the effects of these factors on average and high costs, including total costs and OOP expenditures. The total costs contain all medical expenditure occurred during the hospitalization, which consist of drug fee, bed fee, nursing fee, etc. The OOP expenditures are the actual direct medical expenses paid by inpatients after reimbursement of medical insurance. For example, the expenditure of a TB inpatient is 200 USD, and the reimbursement ratio is 70%, then the OOP is 140 USD (200 USD*70%). The reimbursement ratio is often determined by medical insurance department or health administration department according to number of funds, economic level, number of insured residents, etc. [18] The indirect costs during hospitalization (such as transportation, room and board costs) nor the opportunity costs (costs of care, charge for loss of working time, etc.) are not included in OOP. The total costs can reflect the services supply behaviors, and OOP can reflect the compensation level of medical insurance and economic burden for patients to a certain extent. The economic perspective of this study was mainly from patients, as we concentrated on the direct medical economic burden to inpatients costed by the health services during hospitalization. Through our analysis, we expect to provide targeted advices on the management of hospitals and optimization of the compensation policy of medical insurance to decrease the economic burden on rural TB inpatients.

## Methods

### Study area

**Fig. 1:**
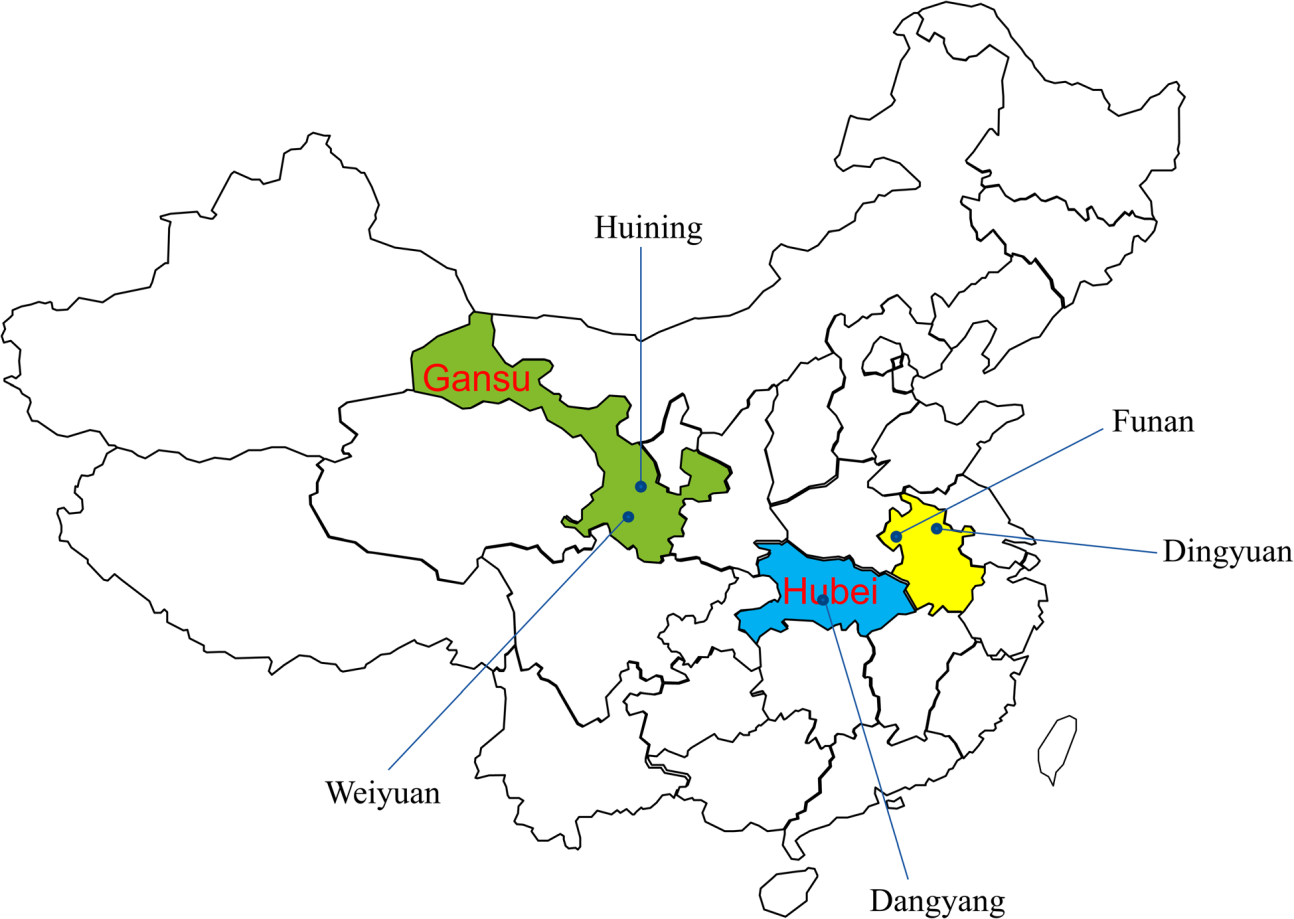
The geographical locations of the sample areas (The map was drawn and labelled by the authors according to the map of China and the location of each sample county)

In this study, five counties (Dangyang, Dingyuan, Funan, Huining, and Weiyuan) were selected as the samples through convenience sampling. The health administrations of these counties have been collaborating with our research team for many years. All counties are located in central or western China, of which the economic development is not as good as that of east China. Dangyang is within the jurisdiction of Yichang City and is located in the central area of Hubei Province. Dingyuan and Funan are located in Anhui Province, whereas Dingyuan is located in the east of Anhui while Funan is located in the northwest. Huining and Weiyuan belong to Gansu Province, which is located in western China. Figure 1 shows the geographic positions of the samples. Table 1 presents the population and economic conditions of the samples in 2016. Each sample county has a large rural population. The constituent ratio of the rural population is over 60% in Dingyuan, over 70% in Dangyang and Huining, and over 90% in Funan and Weiyuan. The per capita disposable household income of rural residents in Dangyang (2678.4 USD) is higher than that in other sample counties. The per capita disposable household income of rural residents in Huining (946 USD) and Weiyuan (944.7 USD) are the lowest.

**Table 1:** Population and economic conditions of the samples (2016)

	Population (ten thousands)	Rural population (ten thousands)	Per capita disposable household income of rural residents (USD)
Dangyang	48.6	35.8	2678.4
Dingyuan	97.5	64.6	1610.9
Funan	172.0	158.8	1404.4
Huining	53.8	38.7	946.0
Weiyuan	34.5	32.2	944.7

### Data collection and outcome measures

The perspective of this study was from patients. Data for this analysis were obtained from the NRCMS settlement database in the five counties from 2014 to 2016. Billing records of all the inpatients that were definitively diagnosed with TB (as the first diagnosis) were extracted from the database. After extracting cases with key information missing (such as the name of medical institution), a total of 3317 TB inpatients were taken into analysis, including drug-resistant inpatients. Inpatients’ medical certificate numbers, genders, ages, family attributes (the “poor” family or the “general” family were assessed by the local government on the basis of the family’s economic situation), admission and discharge dates, admission and discharge diagnoses, total costs, lengths of stay, and hospitalization institutions could be obtained directly from the database. The ages of inpatients were divided into five groups based on the recent age segmentation in China, that is, “children (0–14), youth (15–45), middle-aged (45–59), the young-old (60–74) and the old (≥75)”.

We generated and grouped the institutions following their names and online introduction. First, as first-grade institutions, such as community health service centers, could not treat TB in China, two levels of the institutions were grouped: secondary (more than 100 beds) and tertiary (more than 400 beds) hospitals. Second, two groups were generated based on the institution ownership: private and public groups. Public medical institutions are funded and managed by the government, while private medical institutions are generally invested by enterprises and supervised by the government. Third, two groups were generated on the basis of the functional attributes of the institutions: specialized and general groups. General hospitals do not limit the types of patients and diseases, with a certain number of beds and relatively complete departments. Specialized hospitals generally have certain limits on the range of diseases for admission, and these hospitals are in a leading position in diagnosis and treatment in this professional field. Fourth, provincial attributes (provincial or cross provincial) were clarified in accordance with the consistency between the province where the inpatient lives and the province where the hospital is located.

Total costs and OOP expenditures were also obtained directly from the NRCMS database and selected as the main outcome variables, which were key indicators to evaluate the direct medical economic burden to inpatients covered by medical insurance [19, 20]. As the NRCMS has covered almost all rural residents in China and the database is relatively complete and accurate, our data were representative in indicating the characteristics of TB hospitalization costs in rural China.

### Statistical methods

Shapiro–Wilk test was adopted for the normality test of the distribution of hospitalization costs and OOP expenditures. On this basis, descriptive statistical analysis was conducted through parametric or non-parametric test. Kernel Density Estimations were performed to display the distribution of total costs and OOP among different groups.

Generalized linear models (GLMs) were applied to evaluate the influencing factors. GLMs, introduced by Nelder and Baker (1972), extend linear regression to models with a non-Gaussian or even discrete response. With logarithmic link function and gamma distribution for cost, GLMs are widely used in medical and economic areas [21, 22].

Quantile regression (QR) was further utilized to explore the different effect size predictions of impacting factors. QR, introduced by Koenker and Bassett (1978), is an extension of classical least squares estimation of conditional mean models to the estimation of an ensemble of models for several conditional quantile functions [23, 24]. QR has been widely used in analyzing cost or expenditure in numerous areas and can describe the relationships between the explanatory variables and costs across the entire distribution by enabling the modeling of any conditional quantile of the outcome variable [25]. Moreover, QR does not assume the normality or homoskedasticity of the distribution of hospitalization cost.

Descriptive analyses were conducted using SPSS 19.0. GLMs and QR were performed using Stata 14.0. Prior to statistical analysis, total hospitalization costs and OOP expenditures were adjusted in accordance with the consumer price index (CPI) in 2016 of the city where each county belongs to, to produce further reliable analysis results. Table 2 shows the CPI change in each city.

**Table 2:** Change of CPI in the 5 cities that 5 counties belong to (2014–2016)

County	City	Increase of CPI	
		2015–2014	2016–2015
Dangyang	Yichang	1.0%	2.3%
Dingyuan	Chuzhou	0.8%	1.7%
Funan	Fuyang	1.8%	1.5%
Huining	Baiying	0.3%	1.6%
Weiyuan	Dingxi	1.6%	1.9%

## Results

### Descriptive statistics

Cases from Dangyang, Dingyuan, Funan, Huining, and Weiyuan accounted for 24.22, 17.96, 31.55, 22.92, and 3.35% of the total sample, respectively. The number of male TB inpatients was more than that of female inpatients. Inpatients aged 15–45 and 60–74 years old were more than the other three groups. TB inpatients in the sample areas were hospitalized mainly in public or general hospitals.

**Fig. 2:**
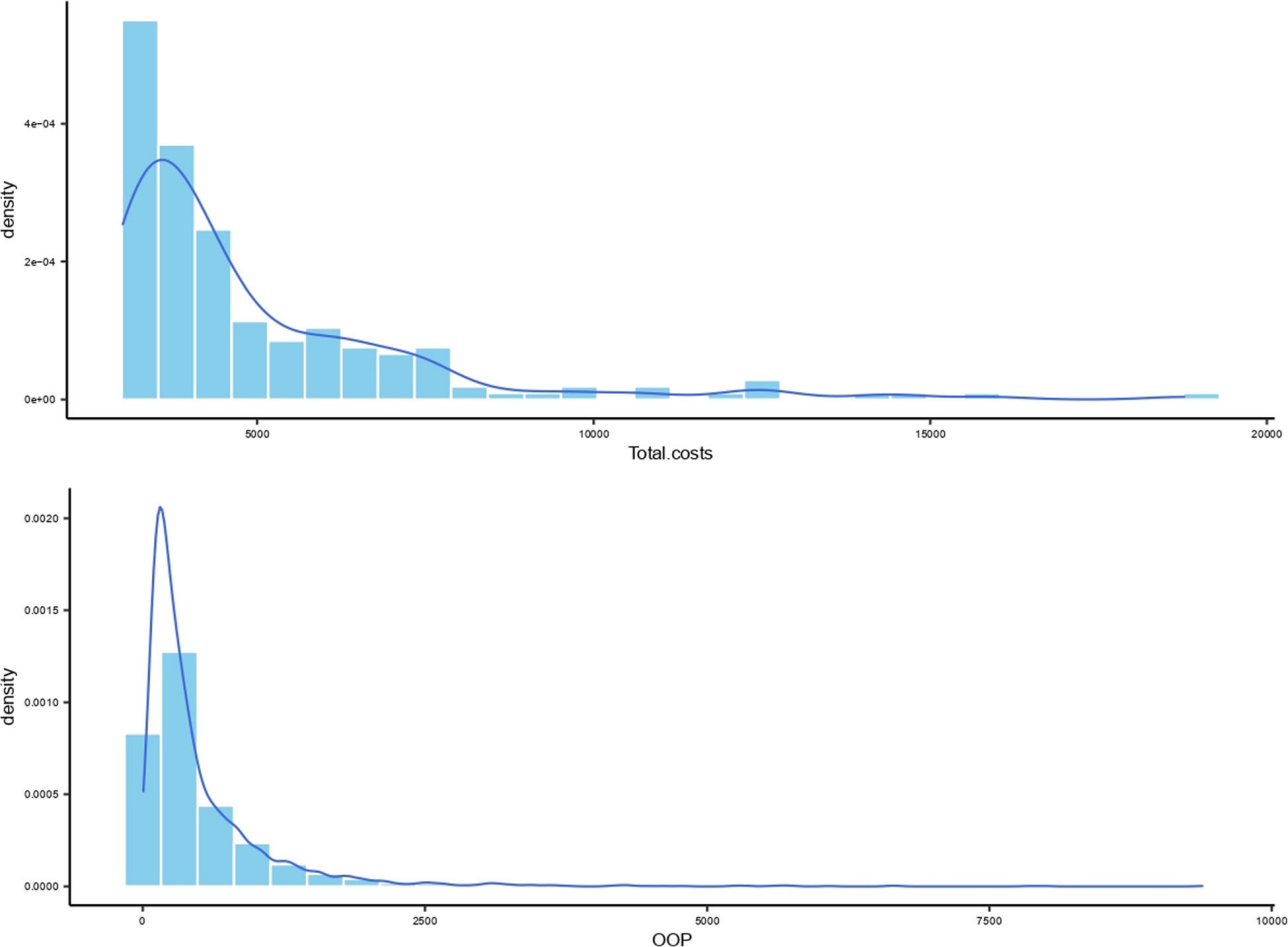
The distribution of total costs and OOP through Kernel Density Estimation

**Fig. 3:**
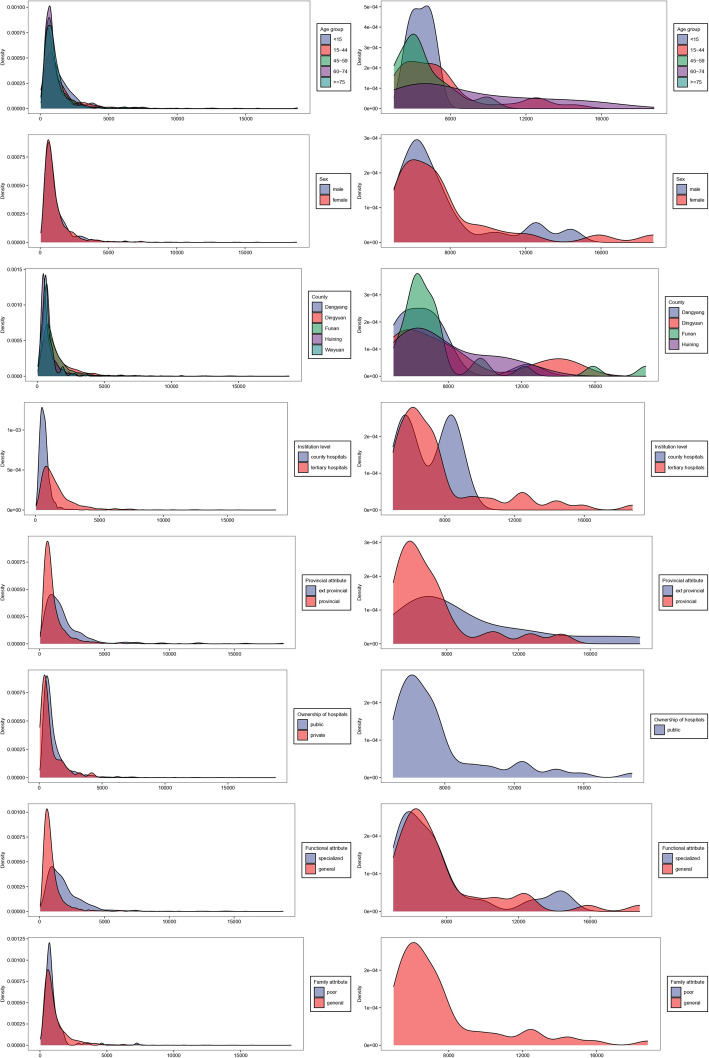
The distribution of total costs among different groups through Kernel Density Estimation

**Fig. 4:**
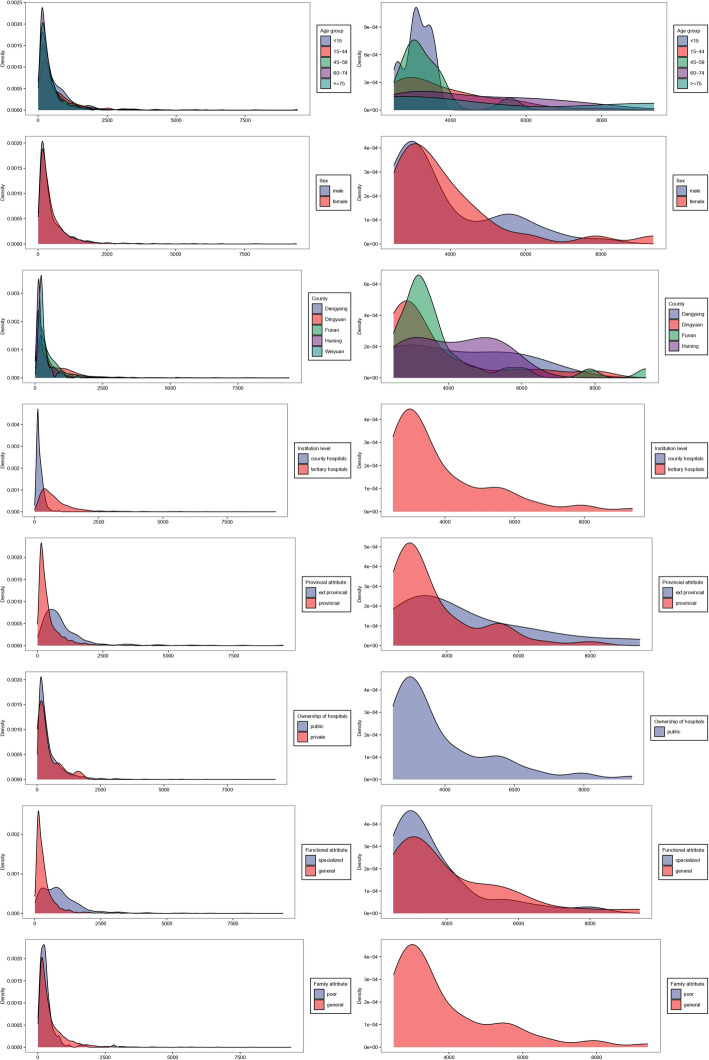
The distribution of OOP among different groups through Kernel Density Estimation

Shapiro–Wilk test showed that total costs and OOP expenditures were in skewed distribution, as skewness values were 4.81 and 4.63, respectively (*p* < 0.05). Therefore, the median was used to describe the sample and nonparametric method was applied to test if statistical differences exist across different groups. Table 3 shows that substantial statistical differences of total hospitalization costs existed across groups based on county, age, institution level, ownership of hospitals, and functional attribute. For OOP expenditures, considerable differences existed among all variables except for sex and ownership of hospitals. The median of total costs in Dingyuan (1087.53 USD) were the highest, whereas OOP expenditures in Funan (410.50 USD) were the highest. The medians of total costs and OOP expenditures in Huining were the lowest (592.73 and 168.10 USD, respectively). Compared to Table 1, the median OOP expenditure in Danyang was 9% of the regional per capita household income, and 23%, 29%, 18% and 25% in Dingyuan, Funan, Huining and Weiyuan, respectively. The highest median of total costs existed in the group of < 15, similar to OOP expenditures. Medians of total costs and OOP expenditures of cross provincially hospitalized inpatients were higher than that of provincially. The median of total costs in tertiary hospitals was higher (1278.24 USD, *p* < 0.001) than secondary hospitals, and that in specialized (1548.37 USD, *p* < 0.001) hospitals was higher than in public hospitals (822.40 USD, *p* < 0.01). OOP expenditures of poor TB inpatients were lower than general inpatients. Table 3 presents additional details. The distributions of total costs and OOP were shown in Figs. 2, 3 and 4, through Kernel Density Estimation.

**Table 3:** Characteristics and hospitalization costs (USD) of tuberculosis inpatients^+^

	Number of inpatients (n)	Percentage (%)	Median of total costs	Median of OOP expenditures
**Sex**				
male	1883	59.53	821.70	290.07
female	1280	40.47	816.21	320.71
**Age**			***	***
< 15	181	5.72	1029.85	443.43
15–44	939	29.69	869.78	340.01
45–59	685	21.66	811.06	308.23
60–74	949	30.00	773.28	261.21
≥ 75	409	12.93	832.14	280.94
**County**			***	***
Dangyang	766	24.22	746.22	256.06
Dingyuan	568	17.96	1087.53	372.29
Funan	998	31.55	993.36	410.50
Huining	725	22.92	592.73	168.10
Weiyuan	106	3.35	649.99	237.54
**Length of stay**			***	***
< 5	470	14.86	693.35	313.38
5–7	541	17.10	738.10	287.75
8–10	508	16.06	733.25	257.46
> 10	1644	51.98	891.52	319.73
**Provincial attribute**			***	***
Ext provincial	283	8.95	1396.39	723.47
Provincial	2880	91.05	790.27	278.10
**Institution level**			***	***
County hospitals	1682	53.18	637.89	169.94
Tertiary hospitals	1481	46.82	1278.24	632.56
**Ownership of hospitals**			**	
Public	3124	98.77	822.40	301.69
Private	39	1.23	573.29	263.65
**Functional attribute**			***	***
Specialized	573	18.12	1548.37	813.59
General	2590	81.88	742.97	258.58
**Family attribute**				**
Poor	117	3.70	748.13	254.46
General	3046	96.30	823.01	304.01

^+^① Note: *p < 0.1; **p < 0.05; ***p < 0.001

② Explanations of the variables:

Family attributes: the “poor” family or the “general” family assessed by the local government on the basis of the family’s economic situation)

Ownership of hospitals: “Public” medical institutions are invested and managed by the government, while the “private” are generally invested by enterprises and supervised by the government

Functional attribute: “Specialized” hospitals generally have certain limits on the range of diseases admitted, while the “general” hospitals do not limit the types of patients and diseases

Provincial attribute: The “provincial” inpatients get medical services within the province of residence, while the “Ext provincial” get medical services out of the province of residence

The reimbursement rates were distributed non-normally. As Table 4 shows, statistical differences exist among all the comparison groups. The higher reimbursement rates existed within groups of Dangyang and Weiyuan Counties, provincial inpatients, county hospitals, public hospitals and general hospitals.

**Table 4:** Description of reimbursement rates in sample counties^+^

	Mean	Median	*P*-value
**County**			< 0.001
Dangyang	63.44	62.23	
Dingyuan	56.56	57.64	
Funan	56.96	54.00	
Huining	62.03	69.05	
Weiyuan	59.25	60.61	
**Provincial attribute**			< 0.001
Ext provincial	46.96	50.00	
Provincial	60.95	62.24	
**Institution level**			< 0.001
County hospitals	68.88	70.83	
Tertiary hospitals	49.26	50.00	
**Ownership of hospitals**			0.001
Public	59.79	59.54	
Private	52.24	50.00	
**Functional attribute**			< 0.001
Specialized	50.86	50.00	
General	61.65	62.35	

^**+**^The statistical variances among different groups were obtained by Mann-Whitney U test

### Results of generalized linear models

The GLMs estimated that the total costs and OOP expenditures in Dingyuan were higher than those in Danyang, and those in Huining were lower than those in Dangyang. TB inpatients in tertiary hospitals had higher average total costs and OOP expenditures (*p* < 0.001) than those in secondary hospitals. TB inpatients in specialized hospitals incurred an average of 350.96 USD increase (*p* < 0.001) in the total cost and 203.68 USD increase (*p* < 0.001) in OOP expenditures compared to general hospitals. The total costs in private hospitals were lower than public hospitals, however, no significant difference existed in OOP between them. Patients with longer length of stay had higher total costs and OOP. (*P* < 0.001). Table 5 shows the details.

**Table 5:** Final generalized linear model results for the variables associated with TB inpatients’ hospitalization costs and OOP expenditures^+^

	Total costs			OOP expenditures		
	β	95% CI	*P*-value	β	95% CI	*P*-value
**Constant**	265.35	(− 429.66, 960.37)	0.454	−208.00	(− 561.84, 145.84)	0.249
**Sex**	0.87	(−78.41, 80.16)	0.983	−0.73	(−41.10, 39.63)	0.972
**Age**						
15–44	−79.03	(− 260.10, 102.05)	0.392	−25.03	(− 117.21, 67.16)	0.595
45–59	− 164.31	(− 349.067, 20.44)	0.081	−80.42	(− 174.47, 13.64)	0.094
60–74	−96.27	(− 275.96, 83.42)	0.294	−57.85	(− 149.33, 33.63)	0.215
≥ 75	− 114.72	(−311.79, 82.35)	0.254	−64.00	(−164.33, 36.33)	0.211
**County**						
Dingyuan	276.72	(148.71, 404.73)	< 0.001	134.45	(69.28, 199.62)	< 0.001
Funan	190.27	(66.95, 313.59)	0.002	16.38	(−46.41, 79.16)	0.609
Huining	− 250.73	(− 365.98, − 135.47)	< 0.001	−156.02	(−214.69, −97.34)	< 0.001
Weiyuan	−114.13	(− 336.61, 108.35)	0.315	−8.34	(− 121.61, 104.93)	0.885
**Length of stay**	34.76	(31.50, 38.01)	< 0.001	13.85	(12.19, 15.50)	< 0.001
**Provincial attribute**	− 540.94	(− 684.58, − 397.31)	< 0.001	− 359.98	(− 433.11, − 286.86)	< 0.001
**Institution level**	706.70	(618.28, 795.13)	< 0.001	494.26	(449.24, 539.28)	< 0.001
**Ownership of hospitals**	− 405.83	(− 753.50, −58.17)	0.022	−141.87	(− 318.88, 35.13)	0.116
**Functional attribute**	− 325.39	(− 437.18, − 213.60)	< 0.001	− 191.90	(− 248.81, − 134.98)	< 0.001
**Family attribute**	119.17	(−90.86, 329.19)	0.266	95.16	(−11.76, 202.09)	0.081

^**+**^ The references groups: Sex: male; Age: 0–14; County: Danyang; Provincial attribute: cross provincial; Institution level: secondary hospitals; Ownership of hospitals: public; Functional attribute: specialized; Family attribute: poor

### Results of quantile regressions

QR analysis estimated the different effect sizes of each factor on different cost segments. For total medical costs, TB inpatients in Huining and Weiyuan were associated with lower expenditures than Danyang at all percentiles. Total expenditures were higher in Dingyuan at all percentiles and in Funan at the 50th, 75th and 90th percentiles compared to Dangyang. (Table 6). Differences within ownership of hospitals and provincial attribute was more substantial at the 25th, 50th and 75th percentiles and the differences disappeared in 90th percentile.

**Table 6:** Quantile regression results of TB inpatients’ total hospitalization costs^+^

	25th percentile		50th percentile		75th percentile		90th percentile	
	β	95% CI	β	95% CI	β	95% CI	β	95% CI
**Constant**	826.08^***^	(586.63, 1065.54)	732.40^***^	(352.62, 1112.18)	711.17^***^	(147.78, 1274.56)	20.41	(− 1423.85, 1464.67)
**Sex**	1.92	(−22.73, 26.58)	−14.11	(−46.24, 18.02)	1.29	(−41.13, 43.71)	−0.08	(−57.60, 57.44)
**Age**								
15–44	−59.43	(− 196.42, 77.56)	−148.92^**^	(−278.56, −19.29)	− 242.05^**^	(− 475.81, −8.29)	− 281.65	(− 712.69, 149.40)
45–59	−75.38	(− 197.37, 46.61)	− 120.13^*^	(−248.00, 7.74)	− 205.12^*^	(− 428.16, 17.92)	−329.95	(− 734.37, 74.46)
60–74	− 41.11	(− 173.26, 91.04)	−76.93	(−214.25, 60.38)	− 184.41	(− 425.28, 56.47)	− 264.36	(− 667.74, 139.03)
≥ 75	−29.41	(−156.54, 97.72)	−71.21	(− 216.86, 74.44)	− 118.97	(− 347.27, 109.33)	− 183.45	(− 594.76, 227.85)
**County**								
Dingyuan	125.75^***^	(78.05, 173.44)	193.05^***^	(151.51, 234.60)	217.22^***^	(170.59, 263.84)	233.09^***^	(153.97, 312.22)
Funan	−36.68	(−98.84, 25.49)	155.88^***^	(75.85, 235.92)	403.00^***^	(293.02, 512.97)	676.81^***^	(531.61, 822.01)
Huining	− 181.60^***^	(−213.59, −149.61)	− 238.88^***^	(− 271.19, −206.57)	− 289.82^***^	(−336.86, −242.77)	− 313.06^***^	(− 389.69, − 236.43)
Weiyuan	−116.01^***^	(− 169.43, − 62.59)	− 118.38^***^	(− 177.36, − 59.39)	−134.49^**^	(− 231.25, − 37.73)	−174.02^**^	(−300.74, − 47.30)
**Length of stay**	18.96^***^	(15.00, 22.91)	33.49^***^	(29.86, 37.12)	46.44^***^	(41.24, 51.63)	54.75^***^	(47.42, 62.07)
**Provincial attribute**	−163.13^***^	(− 259.66, −66.59)	− 245.12^***^	(−392.86, − 97.37)	− 468.96^***^	(− 725.47, − 212.45)	− 752.78	(− 1795.50, 289.95)
**Institution level**	274.35^***^	(227.59, 321.12)	464.50^***^	(422.07, 506.94)	666.80^***^	(594.07, 739.53)	1185.34^***^	(914.63, 1456.05)
**Ownership of hospitals**	− 313.40^***^	(− 385.87, − 240.93)	−347.56^***^	(−425.49, − 269.64)	− 388.51^**^	(− 504.52, − 272.50)	−321.85	(− 768.02, 124.31)
**Functional attribute**	−249.46^***^	(− 349.41, − 149.50)	− 291.62^***^	(−405.00, − 178.25)	−410.78^***^	(− 522.01, − 299.55)	−468.87^***^	(− 600.28, − 337.45)
**Family attribute**	1.37	(−72.41, 75.15)	− 41.21	(− 95.03, 12.61)	5.96	(−69.71, 81.63)	16.29	(−121.81, 154.39)

^**+**^① Note: *p < 0.1; **p < 0.05; ***p < 0.001

② The reference groups: Sex: male; Age: 0–14; County: Danyang; Provincial attribute: cross provincial; Institution level: secondary hospitals; Ownership of hospitals: public; Functional attribute: specialized; Family attribute: poor

Table 7 suggests that patients aged more than 15 tended to have less OOP payments at the 50th and 75th percentiles, but age’s disparities in OOP was non-statistically significant at 25th and 90th percentiles. The OOP in Dingyuan at 75th and 90th percentiles were not higher than Dangyang. TB inpatients in Funan had no higher OOP payments than those in Danyang at the 50th,75th and 95 percentiles. Inpatients in Huining were associated with lower OOP than those in Danyang at all percentiles, while lower in Weiyuan at 50th and 75th percentiles. Compared to patients hospitalized in public hospitals, patients stayed in private hospitals may have the less OOP payments at 90th percentile. But this difference disappeared in other three percentiles.

**Table 7:** Quantile regression results of TB inpatients’ OOP expenditures^+^

	25th percentile		50th percentile		75th percentile		90th percentile	
	β	95% CI	β	95% CI	β	95% CI	β	95% CI
**Constant**	−11.19	(− 146.70, 124.32)	72.89	(− 102.51, 248.30)	110.70	(−179.43, 400.84)	− 165.73	(− 1019.23, 687.76)
**Sex**	1.38	(−10.02, 12.77)	−0.95	(− 15.41, 13.50)	5.26	(−8.22, 18.73)	10.52	(−11.34, 32.38)
**Age**								
15–44	−9.87	(−40.13, 20.38)	− 82.49^***^	(− 132.96, − 32.02)	−91.16^*^	(− 185.67, 3.35)	− 81.30	(− 267.69, 105.10)
45–59	− 4.13	(− 35.83, 27.57)	− 80.60^***^	(− 129.42, − 31.78)	− 93.55^**^	(− 178.10, − 9.01)	−113.70	(− 295.32, 67.93)
60–74	− 5.16	(− 37.40, 27.08)	− 81.45^**^	(− 131.87, − 31.03)	− 82.02^*^	(− 169.12, 5.08)	− 88.98	(− 263.84, 85.88)
≥ 75	−9.09	(−42.05, 23.87)	−61.57^**^	(− 122.12, −1.01)	−74.36	(− 166.05, 17.34)	− 71.19	(− 260.73, 118.34)
**County**								
Dingyuan	103.04^***^	(88.13, 117.96)	96.31^***^	(68.46, 124.16)	36.81	(11.05, 62.56)	51.61	(0.60, 102.62)
Funan	−15.41	(−37.76, 6.94)	9.28	(−19.63, 38.19)	4.79	(− 35.00, 44.57)	93.77	(22.15, 165.39)
Huining	−25.82^***^	(− 40.10, −11.53)	− 84.21^***^	(− 107.76, − 60.65)	−165.97^***^	(− 182.03, − 149.90)	− 182.98^***^	(−210.36, − 155.60)
Weiyuan	56.74^***^	(34.57, 78.91)	−0.24	(−26.47, 26.00)	−56.45^**^	(− 93.03, − 19.86)	−45.71	(− 107.06, 15.63)
**Length of stay**	4.87^***^	(3.59, 6.15)	10.90^***^	(9.00, 12.80)	13.84^***^	(11.37, 16.31)	18.06^***^	(12.69, 23.43)
**Provincial attribute**	−133.55^***^	(− 198.89, − 68.22)	−198.19^***^	(− 253.78, − 142.59)	− 349.23^***^	(− 432.80, − 265.67)	− 542.08^**^	(− 996.04, − 88.12)
**Institution level**	242.74^***^	(226.66, 258.81)	355.60^***^	(331.90, 379.30)	522.30^***^	(465.81, 578.78)	846.85^***^	(721.32, 972.37)
**Ownership of hospitals**	−112.28^***^	(−147.37, − 77.20)	−196.23^***^	(− 250.41, − 142.05)	−267.07^***^	(− 351.88, − 182.26)	−336.02^**^	(− 585.65, − 86.39)
**Functional attribute**	−94.75^***^	(−149.43, − 40.08)	−81.47^**^	(− 151.83, − 11.12)	− 103.08^***^	(− 149.64, − 56.51)	−155.75^***^	(− 209.50, − 102.00)
**Family attribute**	18.06^*^	(−3.29, 39.41)	6.90	(−28.17, 41.97)	17.20	(−9.36, 43.77)	30.61^*^	(−4.68, 65.91)

^**+**^① Note: *p < 0.1; **p < 0.05; ***p < 0.001

② The reference groups: Sex: male; Age: 0–14; County: Danyang; Provincial attribute: cross provincial; Institution level: secondary hospitals; Ownership of hospitals: public; Functional attribute: specialized; Family attribute: poor

## Discussion

This study assessed the effect of socio-demographic and institutional factors on TB inpatients’ total costs and OOP expenditures by using GLMs and QR statistical methods. Several main findings were obtained by both models: when the LOS was longer, TB inpatients easily incurred high costs. TB inpatients who sought cross provincial care possibly faced higher costs, especially at 90th percentile. TB inpatients in tertiary or specialized hospitals easily incurred high costs. Total costs and OOP expenditures were the lowest in Huining. In quantile regressions, the coefficient at the higher percentiles of expenditures indicates the association between the impacting factors and high-intensity care (such as the highly intensive cares or expensive high-technology care) [26]. Considerably different results were produced by QR: High total costs (Q75 and Q90) were incurred in Dingyuan and Funan, but they were not accompanied by high OOP expenditures.

In our study, the median OOP expenditure of TB inpatients in the five counties as a percentage of the area’s per capita income was from 9 to 29%, with the lowest in Danyang. In another study about TB inpatients’ economic burden in China, the median OOP of TB inpatients out of the area’s per capita income was 23.3% in Taixing County [27], which was close to those in Dingyuan, Funan and Weiyuan in our study. Compared to a study of Pan H Q (2013), which evaluated the economic burden of TB inpatients in other three counties in Yichang (which Dangyang belongs to), the OOP out of the area’s per capita income in Dangyang was lower than those counties (14.5, 18.8 and 26.4%) [28]. This may be caused by the higher economic level in Danyang county. Besides, Danyang was selected as one of the payment system reform pilots in Yichang City (mentioned in Background), which achieved great effectiveness, reflected in its high reimbursement rates.

Length of stay is an important factor associated with high costs [29]. The TB inpatients with a more than 10 days LOS constituted more than 50% of the whole sample. The long LOS is an indication of great consumption of medical resources. For inpatients, this may also consume a lot of direct costs, indirect costs and opportunity costs [30, 31]. The prolonged length of stay may be associated with the disease severity. In rural China, the management and treatment of TB are not that standard, and the poor-educated rural TB inpatients are lacking in self-management awareness and compliance. This may lead the rural TB inpatients to postpone admission in more severe cases, requiring more time for recovery [32]. This indicates that additional efforts should be exerted to control the TB morbidity in rural China, as well as strengthen the daily health management and improve the quality of outpatient services for TB patients [33].

The high economic burden on TB inpatients getting medical treatments cross provincially is mainly caused by the inadequate reimbursement policies of medical insurance for allopatric medical services. Allopatric medical services means the medical treatment behavior of the enrollees occurred outside the pooling region where the enrollees insured. It mainly includes medical treatments for one-time (business trip, travelling, etc.), middle/ short-term (such as migrant workers) and long-term relocated retirees. The reimbursement rates of allopatric medical services in our study were much lower than that within provinces. Allopatric medical services of TB are mainly for migrant workers, as all sample counties in our study are migratory places with a large number of migrant workers. Migrant workers were reported to have higher TB rates than local residents in China [34, 35]. Until 2016, the policy on the settlement of allopatric medical services by medical insurance has not been well implemented due to the segmentation and poor coordination of basic medical insurance, the distinct disparity across hospitals in different areas, and the diversity of the causes of allopatric medical services [36]. TB treatment brings a high economic burden to migrant TB patients, who often have less knowledge on timely prevention and treatment and have unsteady income [37–39]. Thus, reimbursement policies of medical insurance for allopatric medical services among TB patients should be optimized, especially for migrant workers.

The higher costs incurred by TB inpatients in tertiary hospitals than in secondary hospitals is not difficult to explain, as better treatment techniques and equipment, and lower reimbursement rates of medical insurance are available in tertiary hospitals. Importantly, an increasing number of rural TB inpatients prefer to acquire hospitalization services in high-level hospitals. In our study, the constituent ratio of rural inpatients hospitalized in tertiary hospitals was nearly 50%. Rural residents in China are not restricted in selecting medical institutions, and this randomness often results in irrational choices, which also affects TB patients’ costs. This irrationality is caused by the increase of living standards, health awareness, and the weak capability of medical institutions within counties [40]. As the standard of diagnosis and treatment for TB is faulty and not effectively implemented, the costs related to TB vary across different hospitals. This can also explain why the costs in specialized hospitals were considerably higher than those in general hospitals in our study. Additional efforts should be exerted to regulate the service standardization for TB treatment and encourage TB patients to rationally select medical institutions to acquire medical services. Besides, drug-resistance may be associated with high medical costs of TB inpatients. In a recent study, the pooled prevalence of drug-resistant TB was 20.1% (18.0–22.3%) among new cases and 49.8% among retreatment cases (46.0–53.6%) within the mainland of China [41]. Treatment of drug-resistant TB has high requirements for advanced clinical technology and medical resources with certain level of quantity/quality [41–43]. Thus, the drug-resistant TB may require treatment at more specialized facilities. This situation, together with the lower reimbursement rates, may also in part explain why the higher costs occurs in tertiary and specialized hospitals.

The stable OOP expenditures in Dingyuan and Funan under their high total costs may be due to the payment system in Anhui Province. Anhui implemented a payment system reform in 2015. This reform mainly includes the construction of medical alliance within the county and the global budget. The medical alliance connects the interests of the member hospitals as a whole. The global budget is prepaid to the medical alliance, and they manage the excess part of the budget and share the balance. This case may increase the suppliers’ awareness of reducing avoidable medical services and improving treatment quality. Moreover, drug-resistant TB has been considered a critical disease by the NRCMS in Anhui Province in 2012. The OOP ratio of drug-resistant TB inpatients was restricted under 30%. The low total costs and OOP expenditures in Huining and Weiyuan may be caused by their low economic level. What we cannot ignore is that at Q90, the association between counties and OOP expenditures was unsubstantial except in Huining. Medical insurance was unable to bring down costs; thus, the economic burden on patients remains heavy. We should strengthen the support for high-cost patients through medical insurance and social assistance to avoid the catastrophic health expenditure.

### Limitations

This study has several potential limitations. First, the variables obtained from the NRCMS database were limited. Other factors associated with higher costs of TB, such as disease severity, could not be obtained from the database. Second, as the diagnosis shown in the database is not complete, the drug-resistant TB inpatients, which may be an important part with high costs, could not be screened out. Drug-resistant TB patients could seek for services at general or specialized hospitals, which may introduce confounding. Third, we could not clarify the variations in services content, intensity, and process among different hospitals. Thus, we could not further explain the causes of differences between specialized and general hospitals. Fourth, we could not distinguish the types of TB that inpatients suffered from based on our database; hence, we could not provide policy suggestions for different types of TB. Fifth, the OOP expenditures represented the direct costs of TB inpatients. However, the indirect costs could not be obtained from the database. Thus, the total economic burden of TB inpatients could not be evaluated through our study. This will be evaluated in our next study through TB patients’ survey.

## Conclusions

This study demonstrates that a long length of stay and obtaining medical services in high-grade, cross provincial, and specialized hospitals are risk factors associated with higher total costs and OOP expenditures among TB inpatients. Helping TB inpatients obtain medical services from appropriate hospitals, improving secondary hospitals’ capability to diagnose and treat TB, and standardizing the diagnosis and treatment process of TB across all designated medical institutions are crucial. Furthermore, ameliorating the reimbursement policy for migrant workers who suffer from TB is necessary to reduce their out of pocket expenditures. Our study further demonstrates the practicability of QR models in investigating issues related to costs, especially those involve high costs.

## Data Availability

The datasets generated and/or analyzed during this study are not publicly available due to anonymity policy issues but are available from the corresponding author on reasonable request. Contact information: chenyingchunhust@163.com.

## Abbreviations

TB: Tuberculosis
NRCMS: New rural cooperative medical system
GLM: Generalized linear model
QR: Quantile regression
OOP: Out of pocket
LOS: Length of stay
